# Prediction of bone mass changes after successful parathyroidectomy using biochemical markers of bone metabolism in primary hyperparathyroidism: is it clinically useful?

**DOI:** 10.20945/2359-3997000000154

**Published:** 2019-07-11

**Authors:** Monique Nakayama Ohe, Teresa Cristina Piscitelli Bonanséa, Rodrigo Oliveira Santos, Murilo Catafesta das Neves, Livia Marcela Santos, Marcello Rosano, Ilda Sizue Kunii, Marise Lazaretti Castro, José Gilberto Henriques Vieira

**Affiliations:** 1 Universidade Federal de São Paulo Departamento de Endocrinologia e Metabolismo Escola Paulista de Medicina Universidade Federal de São Paulo São Paulo SP Brasil Departamento de Endocrinologia e Metabolismo, Escola Paulista de Medicina, Universidade Federal de São Paulo (EPM-Unifesp), São Paulo, SP, Brasil; 2 Universidade Federal de São Paulo Departamento de Otorrinolaringologia, Cabeça e Pescoço EPM Unifesp São Paulo SP Brasil Departamento de Otorrinolaringologia, Cabeça e Pescoço, EPM-Unifesp, São Paulo, SP, Brasil

**Keywords:** Biochemical markers of bone turnover, parathyroid-related disorders, DXA, PTH, osteoporosis

## Abstract

**Objective:**

To measure type 1 serum amino-terminal propeptide procollagen (P1NP) and type 1 cross-linked C-terminal telopeptide collagen (CTX) before parathyroidectomy (PTX) in PHPT patients, correlating these measurements with bone mineral density (BMD) changes.

**Subjects and methods:**

31 primary hyperparathyroidism (HPTP) were followed from diagnosis up to 12-18 months after surgery. Serum levels of calcium, parathyroid hormone (PTH) vitamin D, CTX, P1NP, and BMD were measured before and 1 year after surgery.

**Results:**

One year after PTX, the mean BMD increased by 8.6%, 5.5%, 5.5%, and 2.2% in the lumbar spine, femoral neck (FN), total hip (TH), and distal third of the nondominant radius (R33%), respectively. There was a significant correlation between BMD change 1 year after the PTX and CTX (L1-L4: r = 0.614, p < 0.0003; FN: r = 0.497, p < 0.0051; TH: r = 0.595, p < 0.0005; R33%: r = 0.364, p < 0.043) and P1NP (L1-L4: r = 0,687, p < 0,0001; FN: r = 0,533, p < 0,0024; TH: r = 0,642, p < 0,0001; R33%: r = 0,467, p < 0,0079) preoperative levels. The increase in 25(OH)D levels has no correlation with BMD increase (r = -0.135; p = 0.4816). On linear regression, a minimum preoperative CTX value of 0.331 ng/mL or P1NP of 37.9 ng/mL was associated with a minimum 4% increase in L1-L4 BMD. In TH, minimum preoperative values of 0.684 ng/mL for CTX and 76.0 ng/mL for P1NP were associated with a ≥ 4% increase in BMD.

**Conclusion:**

PHPT patients presented a significant correlation between preoperative levels of turnover markers and BMD improvement 1 year after PTX.

## INTRODUCTION

Primary hyperparathyroidism (PHPT) is a common hypercalcemic disorder caused by abnormally increased secretion of parathyroid hormone (PTH) by one or more parathyroid glands. The hallmark of this condition is the presence of high serum calcium levels and high or inappropriate PTH levels. Although the clinical spectrum of PHPT has changed over the years – from a very symptomatic to a less symptomatic condition – reduced bone mineral density (BMD) is present in several affected patients.

The clinical findings of PHPT are related to the occurrence of hypercalcemia and consistently high PTH levels. PTH promotes calcium reabsorption in the distal renal tubule and regulates the conversion of 25(OH)-vitamin D into 1,25(OH)_2_-vitamin D via 1α-hydroxylase stimulation (1). On bone, PTH can exert anabolic effects on trabecular sites and catabolic effects on cortical sites, depending on the secretion levels and duration of exposure to increased levels of this hormone. In PHPT, a condition in which the bone mass is exposed to high PTH levels, cortical bone areas, such as those in the radius, are more affected than trabecular bone sites, such as those in the lumbar spine. The catabolic effect of PTH is related to an increased production of the receptor activator of nuclear kappa-B ligand and the inhibition of osteoprotegerin by osteoblasts, thus stimulating osteoclastic formation, activity, and survival (2). Radiographic signs of PHPT include skull demineralization in a salt-and-pepper pattern, distal clavicle tapering, subperiosteal bone resorption, and development of cysts and brown tumors. These features combined are described as osteitis fibrosa cystica and are rarely seen in developed countries where more subtle forms of skeletal involvement are observed (3). Data from cohort studies suggest an increased risk of all fractures in PHPT, including vertebral fractures, which appears to decline following parathyroidectomy (PTX) (4,5). Bone health is a clinical concern in PHPT patients, especially in those treated conservatively. Recent studies found a significant BMD decrease in patients treated conservatively and a significant positive BMD increase in patients who underwent PTX (6,7). High-resolution peripheral quantitative computed tomography (HRpQCT) analysis has shown improvement in bone microarchitecture, cortical thickness, density, and estimated strength following PTX in female PHPT patients (8).

Bone is a dynamic tissue in a continuous remodeling process in which formation and resorption work together to maintain a healthy turnover. Bone formation and resorption markers, such as serum amino-terminal propeptide of type 1 procollagen (P1NP) and cross-linked C-terminal telopeptide of type I collagen (CTX), respectively, are indirect indicators of bone turnover providing information on bone metabolic status. Hypersecretion of PTH leads to increased bone turnover in most patients and decreased BMD.

The aim of this study was to determine the P1NP and CTX levels before successful PTX in PHPT patients, and to correlate these measurements with BMD changes.

## SUBJECTS AND METHODS

### Study design

This was a retrospective study evaluating preoperative P1NP and CTX levels in PHPT patients undergoing successful PTX.

This investigation was approved by the EPM-Unifesp Ethics Committee (approval no. 618.187) and all patients gave an informed consent prior to their inclusion in the study.

### Patients

From November 2011 to November 2014, a total of 101 PHPT patients underwent PTX at the Federal University Hospital EPM-Unifesp, São Paulo, Brazil. Among these patients, 31 presented enough data and long-term follow-up to be included in this study. All these patients had undergone a successful PTX and were followed up prospectively from their diagnosis of PHPT up to 12 to 18 months after surgery.

The diagnosis of PHPT was based on the presence of hypercalcemia and high or inappropriate PTH levels. The surgical procedure was indicated to all symptomatic patients with PHPT (presence of nephrolithiasis and/or fragility fractures), as well as asymptomatic ones according to the Fourth International Workshop on the Management of Asymptomatic Primary Hyperparathyroidism (9). The exclusion criteria comprised the use of lithium, thiazide diuretics, bisphosphonates, denosumab, teriparatide, glucocorticoids, hormone replacement therapy and/or a diagnosis of familial hypocalciuric hypercalcemia. The occurrence of the latter was excluded by a urinary calcium:creatinine ratio < 0.01 and serum calcium measurements in first-degree relatives.

The patients underwent a traditional PTX with bilateral neck exploration, and the success of the surgery was confirmed by intraoperative PTH measurement. Histopathological findings confirmed the removal of the affected parathyroids in all 31 patients, revealing a parathyroid adenoma in 30 patients and parathyroid hyperplasia in one. All 31 patients presented normal postoperative calcium levels during follow-up.

MDRD (Modification of Diet in Renal Disease) estimates glomerular filtration rates were above 60 mL/min/1.73 m^2^ in all patients except in two, whose MDRD were 45 and 34 mL/min/1.73 m^2^ due to renal impairment associated with PHPT.

### Biochemical measurements

All biochemical measurements were performed upon diagnosis and 12 to 18 months after surgery. Both turnover markers (P1NP and CTX) were measured using commercial kits (Chemiluminescence, Elecsys 2010 analyzers; Roche Diagnostic, Indianapolis, IN, USA). For P1NP, the normal range was 13.8-60.9 ng/mL in postmenopausal women and 13.9-85.5 ng/mL in men, the intra-assay variation was 1.04%, and the interassay variations were 9.2% and 12.5%. For CTX, the normal range was < 0.650 ng/mL in women and < 0.850 ng/mL in men, the intra-assay variation was 0.55%, and the interassay variations were 7.5% and 7.8%.

PTH was measured by immunoelectrochemical assay (Roche, Elecsys 2010 analyzer, USA; normal range 10-65 pg/mL, intra-assay variation 2.38%, interassay variations 6.7% and 7.5%). Serum calcium, albumin, and creatinine were measured with standard automatic assays. Serum ionized calcium was measured with an ion-specific electrode (AVL 9180 Electrolyte Analyzer, AVL Scientific Corp., Roswell GA, USA). Serum 25-hydroxyvitamin D (25(OH)D) was measured by chemiluminescence assay (Roche, Elecsys 2010 analyzer, USA; normal reference value greater than 30 ng/mL, intra-assay variation 4.1%, interassay variations 17.5% and 17%).

### BMD measurements

Measurements of BMD were performed before and 1 year after PTX with dual-energy X-ray absorptiometry (DXA; Hologic QDR 4500, Waltham, MA, USA) at the lumbar spine (L1-L4), total hip (TH), femoral neck (FN), and distal third of the nondominant radius (R33%). We used the BMD, Z-score and T-score values for each site. The Z-score is the number of standard deviations a given measurement differs from the mean for a sex and age-matched reference population. The T-score is the number of standard deviations a given measurement differs from the mean for a normal young adult reference population. The least significant change (LSC) in BMD measurements in the lumbar spine, proximal femur, and R33% were 3.5%, 3.8%, and 3.2%, respectively.

### Statistical analysis

The statistical analysis was performed using the software GraphPad Prism, version 5 (GraphPad Software, Inc., La Jolla, CA, USA). The data are expressed as mean ± standard deviation (SD) or range. Student’s *t* test was performed for the following preoperative and postoperative parameters: ionized calcium, total calcium, PTH, vitamin D, CTX, and P1NP. We used Pearson’s test for correlation analyses of data with normal distribution; data without normal distribution were first normalized and then analyzed with Pearson’s correlation. The authors opted for utilizing the Pearson test after normalizing data to facilitate the understanding by applying one single test. Multiple linear regression analysis was used to estimate the percentage BMD changes in the lumbar spine, TH, FN, R33%, and preoperative biochemical values. The results were considered significant when *p* < 0.05.

## RESULTS

The demographic and clinical characteristics of the study cohort are shown in Table 1. Of the 28 women, 25 were in postmenopausal and all the men were over 50 years. All subjects had biochemical data consistent with the disease and were symptomatic or met at least one criterion for PTX according to the Fourth International Workshop (9). Biochemical data and BMD measurements before and after PTX are shown in Tables 2 and 3, respectively.

**Table 1:** Demographic and clinical characteristics of 31 patients with primary hyperparathyroidism

Patients	31
Age, mean (± SD) years	60 (±9)
Gender, N (%)	
Female	28 (90.3%)
Male	3 (9.6%)
Asymptomatic patients, N (%)	14 (45.1%)
Symptoms, N (%)	
Kidney stones	16 (51.6%)
Brown tumor	1 (3.2%)
Fragility fractures	3 (9.6%)

**Table 2:** Biochemical data of 31 patients with primary hyperparathyroidism before and after successful parathyroidectomy (n = 31)

	Preoperative	Postoperative (12 months)	p
iCa	1.63 (± 0.17)	1.30 (± 0.04)	< 0.0001
tCa	11.6 (± 0.93)	9.4 (± 0.4)	< 0.0001
PTH	265 (± 337)	55 (± 24.6)	< 0.0001
25(OH)D	16 (± 5.9)	33 (± 14.4)	< 0.0001
CTX	1.05 (± 0.79)	0.23 (± 0.17)	< 0.0001
P1NP	119 (± 100)	39 (± 24.5)	< 0.0001

Results are presented as mean (± SD) values.

iCa: ionized serum calcium (1.24-1.40 mmol/L); tCa: total serum calcium (8.5-10.3 mg/dL); PTH: parathyroid hormone (10-65 pg/mL); CTX: cross-linked C-terminal telopeptide of type I collagen (< 0.650 ng/mL); P1NP: serum amino-terminal propeptide of type 1 procollagen (13.8-60.9 ng/mL); 25(OH)D: 25-hydroxyvitamin D (30-60 ng/mL).

**Table 3:** Dual-energy X-ray absorptiometry (DXA) data of 31 patients with primary hyperparathyroidism before and after successful parathyroidectomy

	Preoperative			Postoperative (12 months)		
	
	BMD	T-score	Z-score	BMD	T-score	Z-score
L1-L4	0.860	-1.7 (± 1.2)	-0.3 (± 1.2)	0.925*	-1.1 (± 1.1)	+0.3 (± 1.2)
FN	0.700	-1.4 (± 0.7)	-0.1 (± 0.7)	0.740*	-1.0 (± 0.8)	+0.4 (± 0.8)
TH	0.806	-1.0 (± 0.8)	-0.2 (± 0.7)	0.847*	-0.7 (± 0.9)	+0.3 (± 0.7)
R33%	0.549	-2.6 (± 1.7)	-1.3 (± 1.6)	0.558*	-2.4 (± 1.5)	-1.1 (± 1.4)

Results are presented as mean (± SD).

L1-L4: lumbar spine; TH: total hip; FN: femoral neck; R33%: distal third of the radius.

* P < 0.05 compared with preoperative values.

Using a reference value of 30 ng/mL for vitamin D, all patients except one were found to be vitamin D deficient (data not shown) (10). Cholecalciferol was prescribed for vitamin D replacement before and after surgery for all patients at a minimum dose of 1000 IU/day.

Normal postoperative calcium levels were observed in all 31 patients during follow-up. Four patients presented transiently increased PTH measurements after surgery related to vitamin D deficiency and hungry bone syndrome.

**Figure 1:**
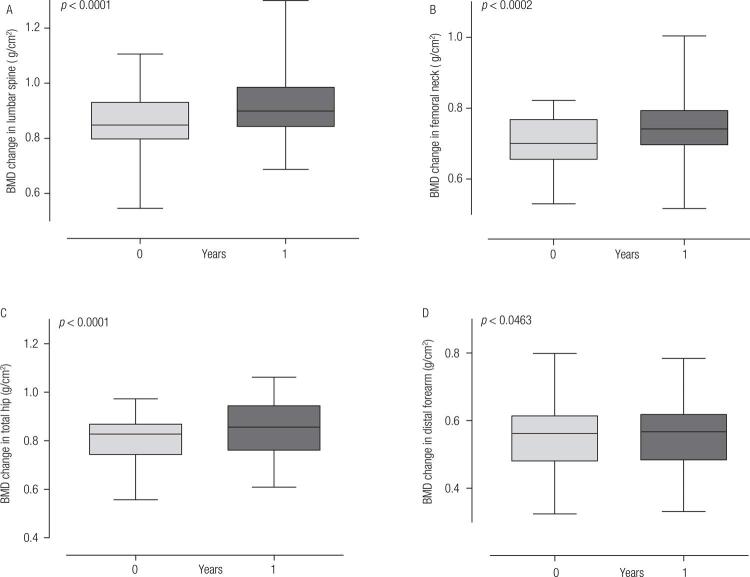
Changes in bone mineral density (BMD; g/cm2) in the lumbar spine, femoral neck, total hip and distal forearm at 1 year after parathyroidectomy.

BMD changes (g/cm^2^) in different bone areas at 1 year after PTX are demonstrated in Figure 1. The mean percentage changes in BMD at 1 year after surgery were 8.6% (*p* < 0,0001), 5.5% (*p* < 0,0002), 5.5% (*p* < 0,0001), and 2.2% (*p* < 0,0463) in the lumbar spine, FN, TH, and distal forearm, respectively.

**Figure 2:**
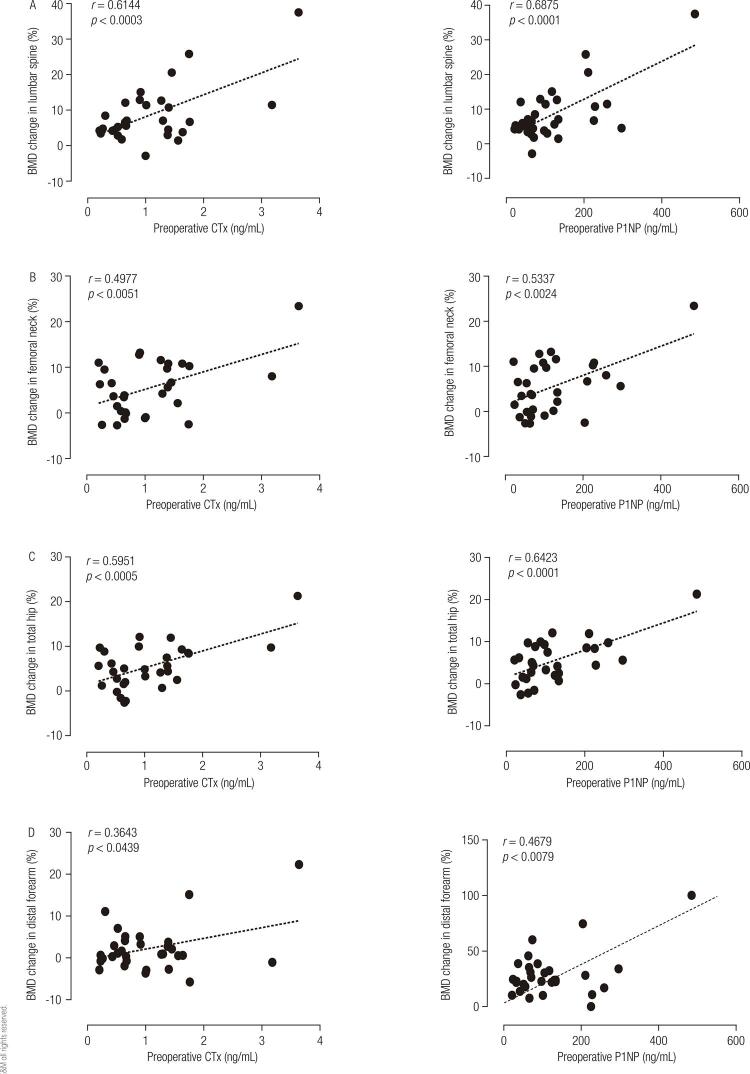
Correlation between the percentage bone mineral density (BMD) change at 1 year after parathyroidectomy and values of preoperative biomarkers in the lumbar spine (A), femoral neck (B), total hip (C), and distal forearm (D).

A significant positive correlation was observed between the percentage BMD change at 1 year after PTX and values of preoperative biomarkers (Figure 2). The increase in 25(OH)D levels has no correlation with the increase in BMD (*r* = -0.135; *p* = 0.481).

On linear regression, a minimum preoperative CTX value of 0.331 ng/mL (y = 6.1786x + 1.9513) or P1NP value of 37.9 ng/mL (y = 0.0547x + 1.9282) was associated with a ≥ 4% increase in lumbar spine BMD.

In TH, minimum preoperative TH values of 0.684 ng/mL for CTX (y = 3.7799x + 1.4218) and 76 ng/mL for P1NP (y = 1.5 + 0.03x) were associated with a ≥ 4% increase in BMD.

Multiple linear regression analysis demonstrated a significant correlation between BMD changes and preoperative values of the biochemical markers of bone metabolism (Table 4).

**Table 4:** Multiple linear regression analysis of the percentage change in bone mineral density (BMD) and biochemical markers of bone metabolism

	Preoperative CTX	Preoperative P1NP
%BMD L1-L4	r2 = 0.3775; p = 0.0003	r2 = 0.4727; p < 0.0001
%BMD FN	r2 = 0.2477; p = 0.0051	r2 = 0.2848; p = 0.002
%BMD TH	r2 = 0.3542; p = 0.0005	r2 = 0.4125; p = 0.0001
%BMD R33%	r2 = 0.1327; p = 0.0439	r2 = 0.2190; p = 0.0079

P1NP: serum amino-terminal propeptide of type 1 procollagen; CTX: cross-linked C-terminal telopeptide of type I collagen; L1-L4: lumbar spine; TH: total hip; FN: femoral neck; R33%: distal third of the radius.

No significant correlation was observed between preoperative P1NP or CTX measurements and 25(OH)D levels (*r* = -0.255; *p* = 0.165 and *r* = -0.193; *p* = 0.298, respectively).

## DISCUSSION

The absence of symptoms has been the dominant clinical phenotype of PHPT in the United States and Western Europe for the past 40 years (11). Even in some developing countries including Brazil, changes in clinical and laboratory findings at the time of diagnosis have been observed over the years, from a very symptomatic condition to an oligosymptomatic and even asymptomatic presentation (12). Many PHPT patients are asymptomatic and have mild hypercalcemia. Their management is still controversial (13). Although overt symptoms are uncommon, PHPT patients still demonstrate involvement of target organs, including nephrolithiasis and skeletal involvement detected by DXA (11).

In the present study, we observed a significant improvement in bone mass 1 year after PTX. The lumbar spine was the region with the most important bone mass gain, with a mean 8.6% change in BMD, followed by the FN and TH, with a mean 5.5% BMD change in both. The forearm presented a mean 2.2% BMD gain 1 year after PTX; although this result showed statistical significance, we considered it to be clinically irrelevant since it does not exceed the least significant change. The present study also demonstrated substantial biochemical changes in both formation (P1NP) and resorption (CTX) markers between the preoperative and postoperative periods in PHPT patients, with a significantly positive correlation between high levels of the preoperative biomarkers CTX and P1NP and BMD change 1 year after successful PTX.

Studies in recent years have looked into bone mass recovery after PTX. Most evidence point to the occurrence of bone damage as being at least a reversible process after a successful surgical procedure (14,15). Nakaoka and cols. have shown bone mass improvement after PTX in asymptomatic patients without surgical indication. In that study, the lumbar spine and forearm presented BMD increases of 12.2% and 11.6%, respectively, after PTX (14). On the other hand, Silverberg and cols. presented results of increased BMD in the lumbar spine and FN of 8.2% and 5.9%, respectively, in PHPT patients (15), which are more aligned with our results. These authors also observed a significant gain of 4% in the forearm only after the third year from the surgical procedure (15). Hesp and cols. confirmed the findings of lack of bone mass gain in cortical sites in the first year after PTX in a patient with parathyroid adenoma, but increased gain at 2 years after the surgery (16). A possible higher bone turnover in trabecular sites may explain the faster BMD recovery observed after PTX than that observed in cortical sites. In physiological conditions, the metabolic rates in trabecular and cortical bone are around 30% and 3%, respectively (17).

Biochemical markers of bone formation and resorption may be an interesting tool in the assessment of bone turnover status in PHPT patients. Markers of bone resorption or formation reflect secretory or breakdown products of bone cells or collagen during bone remodeling process (18). In PHPT, a disorder with increased bone metabolism, these turnover markers may serve as helpful indices of the extent of the influence of the process of hyperparathyroidism on bone (18).

The present study demonstrates substantial biochemical changes in both formation (P1NP) and resorption (CTX) markers before and after PTX (Table 2), and the results are in agreement with those of previous studies (18,19). Recent studies have demonstrated that biochemical resorption markers decrease faster when compared with formation ones, enabling a “window” in which bone formation overcomes resorption (18). A perioperative study investigated the changes in CTX and P1NP two days after PTX in 41 PHPT postmenopausal women. Subjects were divided in groups according to their initial bone markers levels. An increase in P1NP and decrease in CTX levels was observed mainly in patients with higher preoperative bone turnover concentrations. Although BMD was not accessed, it may be suggested that those with higher preoperative bone turnover markers, would have a larger bone gain (20). In our study, we found a significant positive correlation between the percentage BMD change 1 year after PTX and preoperative biomarkers: the greater the preoperative biomarker level, the greater the BMD increase. Alonso and cols. have also demonstrated a positive correlation between preoperative CTX and P1NP levels and postoperative bone mass gain in the lumbar spine 1 year after PTX. Fifty-three PHPT patients were enrolled and followed for one year, but only lumbar spine bone mass was measured (21). In contrast, a six month postoperative study, didn’t find correlation between osteocalcin and urinary pyridinium with BMD gain (20).

This study demonstrated that a minimum preoperative CTX value of 0.331 ng/mL or P1NP value of 37.9 ng/mL was associated with a ≥ 4% increase in lumbar spine BMD. In TH, a minimum preoperative value of 0.684 ng/mL for CTX and 76.0 ng/mL for P1NP was associated with a ≥ 4% increase in BMD. These results point out to a potential role for these bone metabolic markers as predictors of bone mass gain after surgical treatment. Additionally, these bone biomarkers may be useful for surgical recommendation in certain patients with asymptomatic PHPT.

This study has some limitations, mainly related to the small number of patients enrolled and to the important vitamin D deficiency observed among some of these patients before PTX. More studies with a larger number of patients and longitudinal BMD measurements are needed.

In conclusion, there was a significantly positive correlation between BMD changes 1 year after PTX and high preoperative levels of the biomarkers CTX and P1NP in PHPT patients.
